# Supplementary material for: Anti-Leukemic Properties of Aplysinopsin Derivative EE-84 Alone and Combined to BH3 Mimetic A-1210477
Source: Mar Drugs. 2021 May 21;19(6):285. doi: 10.3390/md19060285 (PMC8224038; doi:10.3390/md19060285)
Supplement: Supplementary file 1 [file marinedrugs-19-00285-s001.zip › marinedrugs-1147753-supplementary.pdf]

marine drugs

MDPI

SUPPLEMENTARY MATERIAL

Anti-leukemic properties of aplysinopsin derivative EE-84 alone and combined to BH3 mimetic A-1210477

Sungmi Song [1†], Sua Kim [1†], Eslam R El-Sawy [2,4], Claudia Cerella [1,3], Barbora Orlikova-Boyer [1,3], Gilbert Kirsch [4], Christo Christov [5], Mario Dicato [3] and Marc Diederich [1,*]

[1] Department of Pharmacy, College of Pharmacy, Seoul National University, 1 Gwanak-ro, Gwanak-gu, Seoul, 08626, South Korea; suakim@snu.ac.kr (S.K.); sson35@snu.ac.kr (S.S.)
[2] Chemistry Department of Natural Compounds, National Research Centre, 12622 Dokki, Giza, Egypt; eslamelsawy@gmail.com (E.E.)
[3] Laboratoire de Biologie Moléculaire et Cellulaire du Cancer, Hôpital Kirchberg, 9, Rue Edward Steichen, 2540 Luxembourg, Luxembourg; claudia.cerella@lbmcc.lu (C.Ce.); barbora.orlikova@lbmcc.lu (B.B)
[4] UMR CNRS 7565 SRSMC, Université du Lorraine, 57070 Metz, France; gilbert.kirsch@univ-lorraine.fr (G.K.)
[5] Service d'Histologie, Faculté de Médicine, Université de Lorraine, INSERM U1256 NGERE, 54000 Nancy, France; christo.christov@univ-lorraine.fr
* Correspondence: marcdiederich@snu.ac.kr (M.D.); Tel.: +82-2-880-8919 (M.D.)
† These authors equally contributed to this work

SUPPLEMENTARY TABLE

Table S1. IC_{50} of aplysinopsin analogs in the non-cancerous cell line RPMI 1788. IC_{50} (µM) values were evaluated after indicated times of compound treatment

	**IC_{50} (µM)**		
**Compound**	**24 h**	**48 h**	**72 h**
**EE-84**	> 50	> 50	> 50
**EE-115**	> 50	> 50	> 50

*IC50 values were calculated after trypan blue staining and represent the mean ± S.D. of three independent experiments. IC50 indicates the concentration required to cause 50% cell death.

SUPPLEMENTARY FIGURES

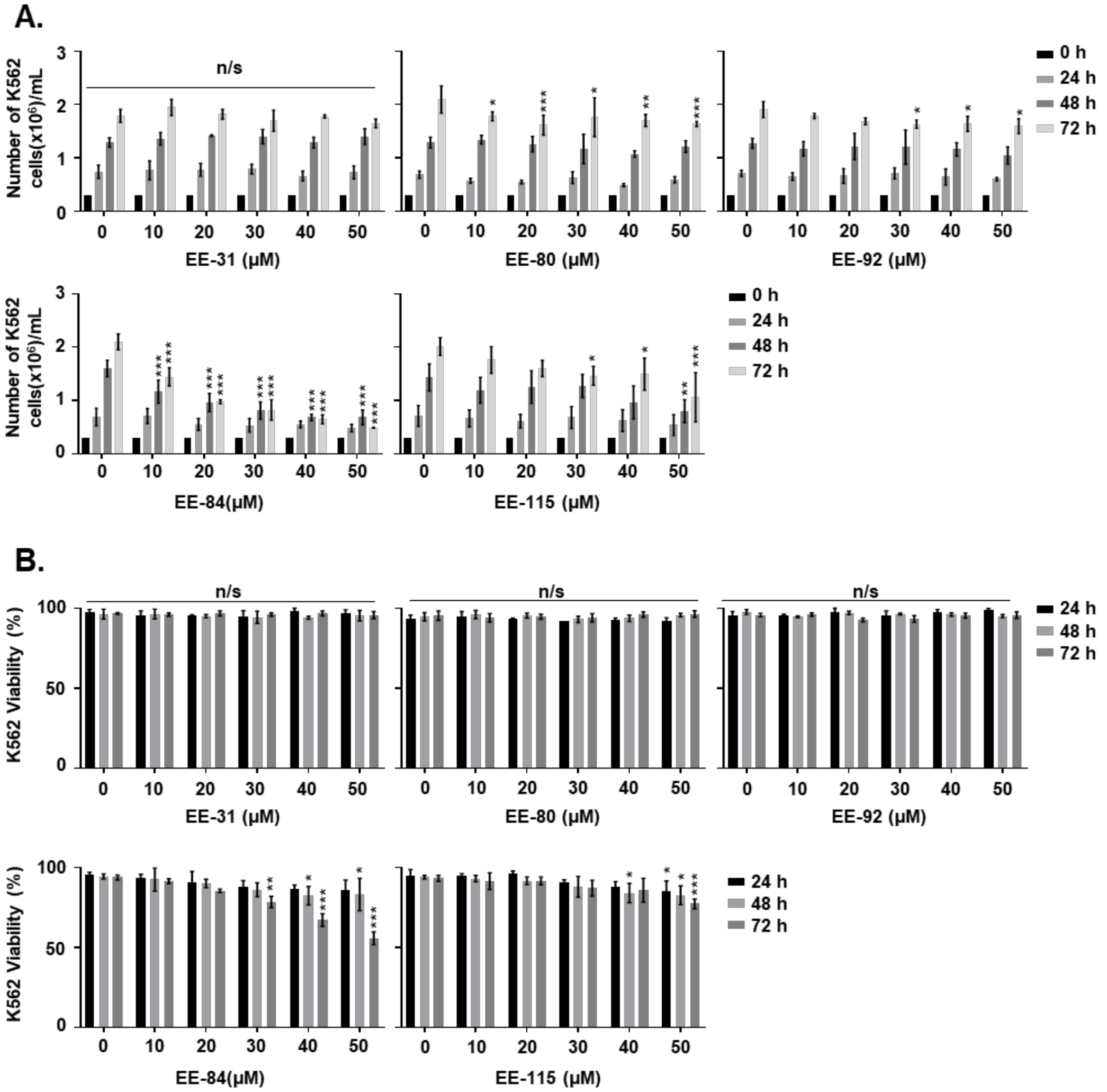

Supplementary Figure 1: Effect on proliferation and viability of aplysinopsin-treated on K562 cells (A) After 24, 48, and 72 h of treatment on K562 cells, (A) cell number and (B) viability were determined by trypan blue exclusion test. Trypan blue exclusion test data represents the mean ± SD of three independent experiments. Statistical analysis: two-way ANOVA with Dunnett's multiple comparison test (trypan blue exclusion test); *p<0.05, **p<0.01, ***p<0.001 compared to controls.

A. ^{1}H NMR spectrum of EE-31.

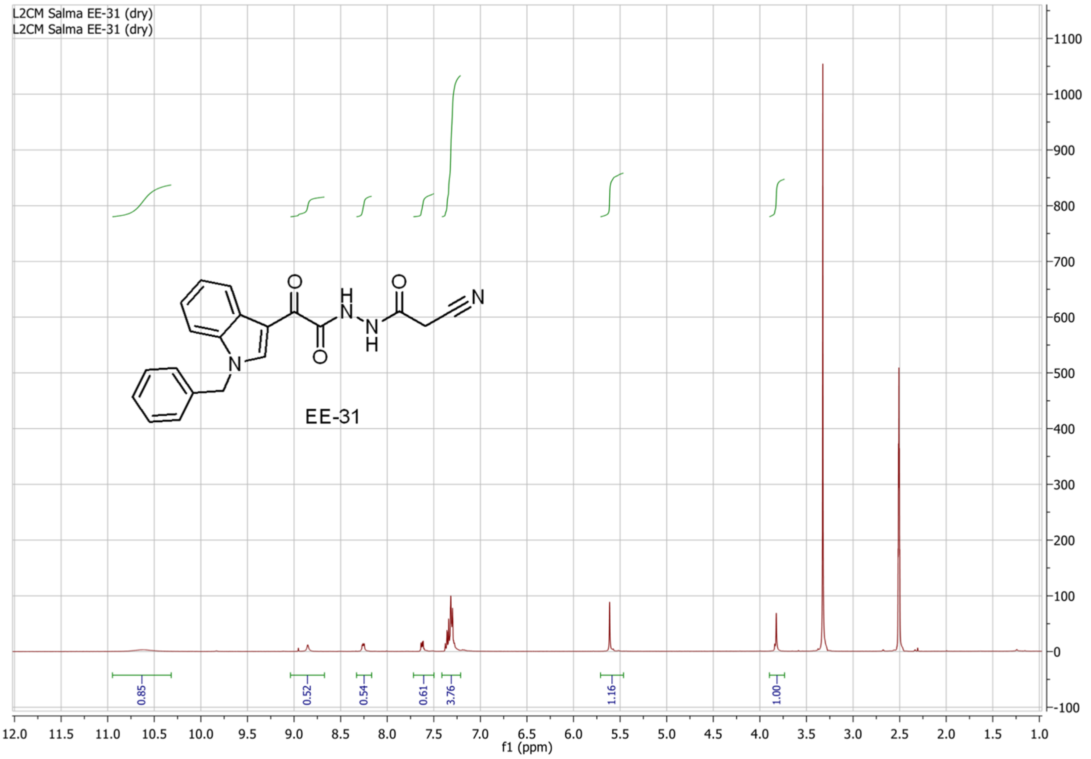

B. ^{1}H NMR spectrum of EE-80.

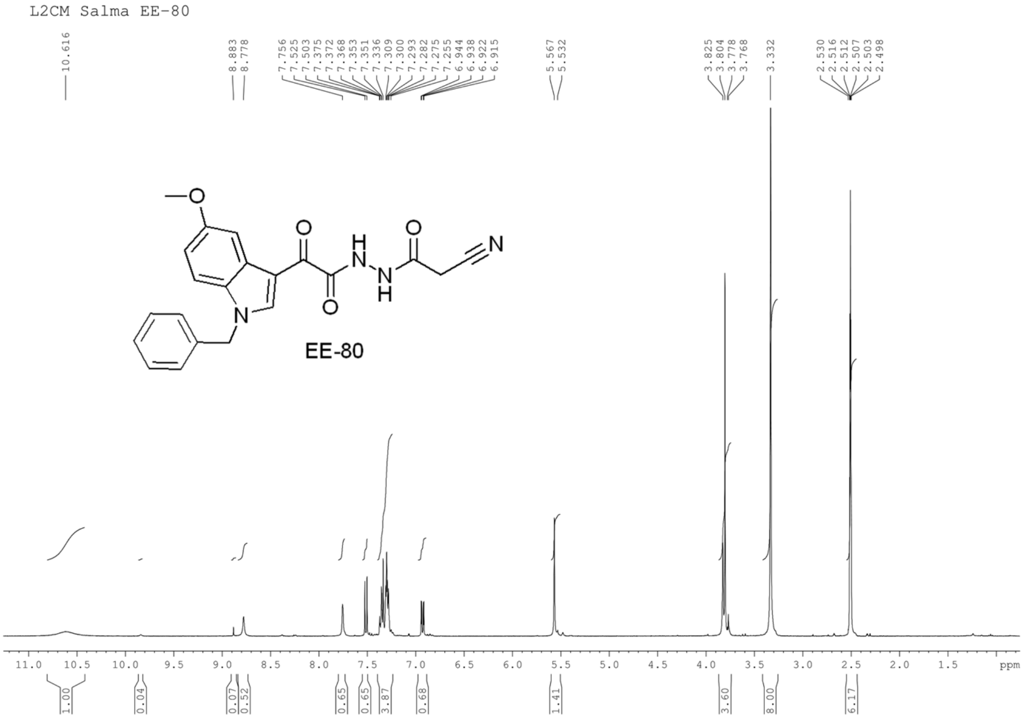

C. ¹H NMR spectrum of EE-84.

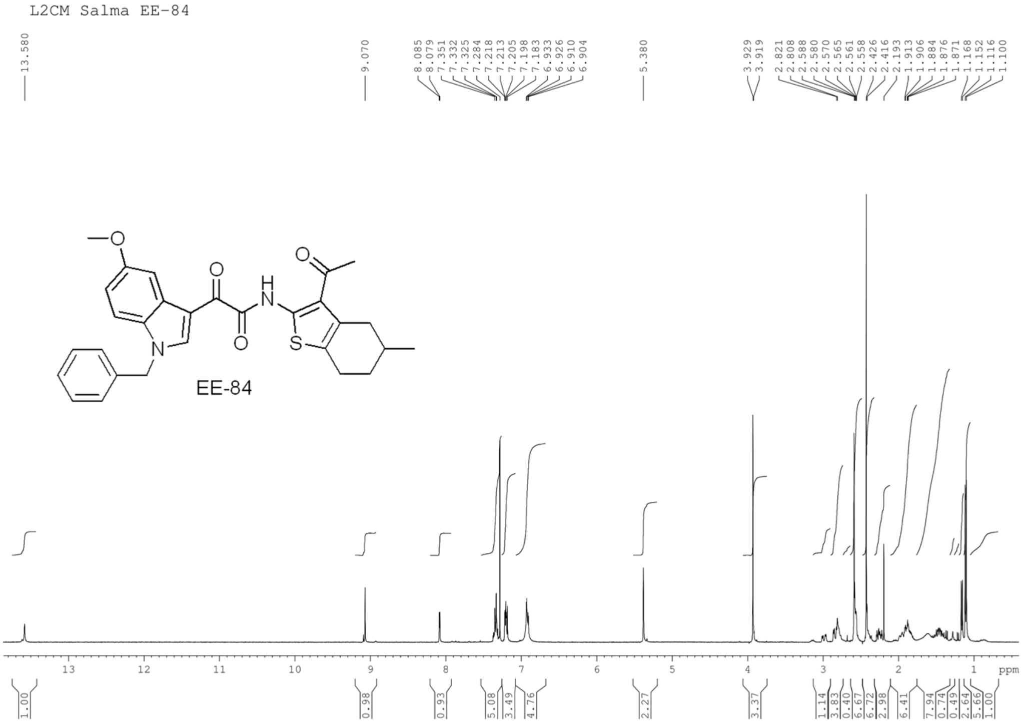

D. ¹H NMR spectrum of EE-92.

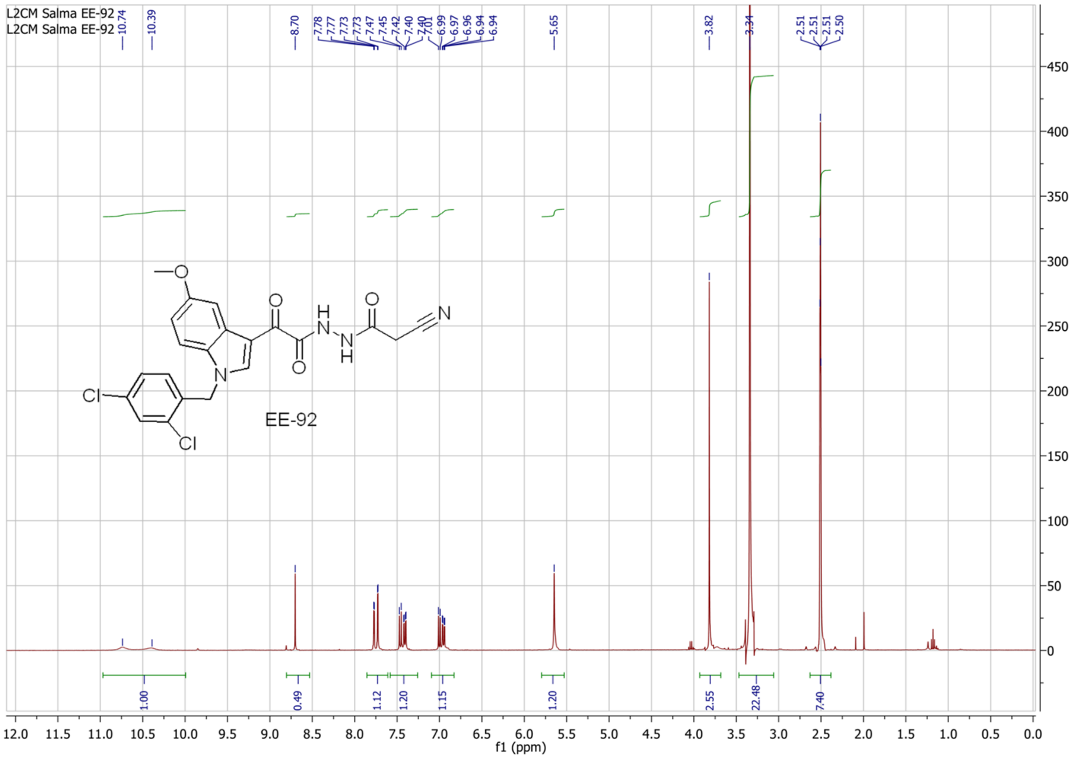

E. ^{1}H NMR spectrum of EE-115.

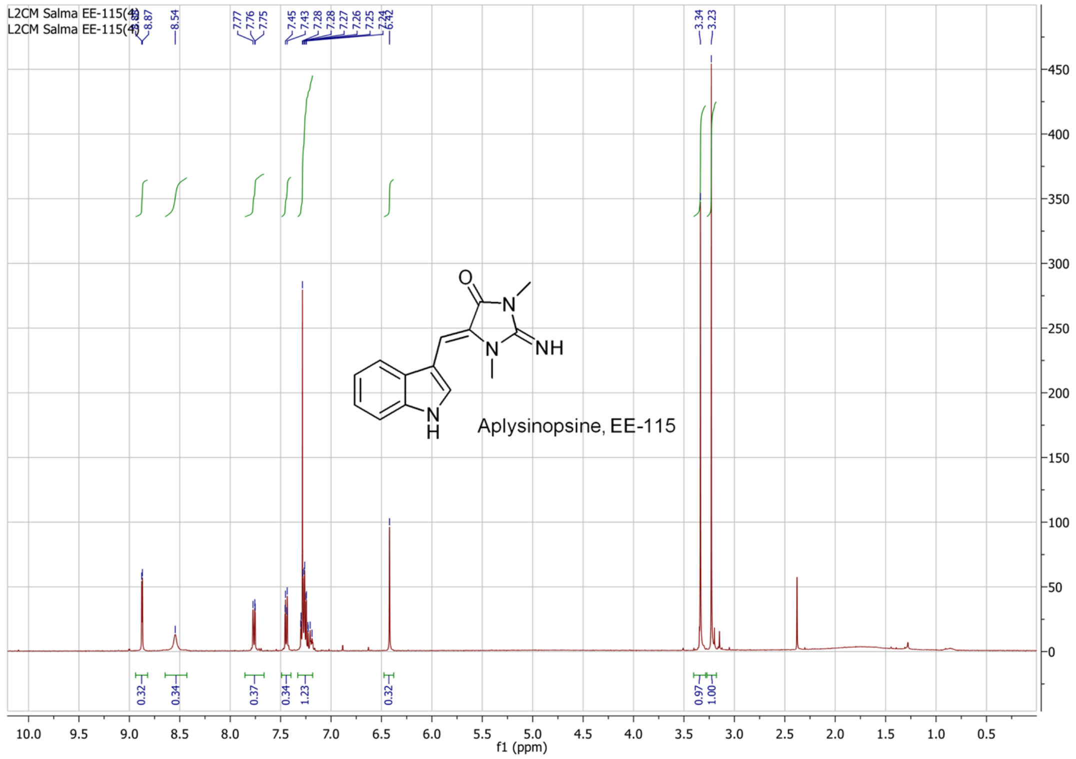

Supplementary Figure 2: ^{1}H NMR spectral data of (A) EE-31, (B) EE-80, (C) EE-84, (D) EE-92 and (E) EE-115.

A. ^{13}C NMR spectrum of EE-31.

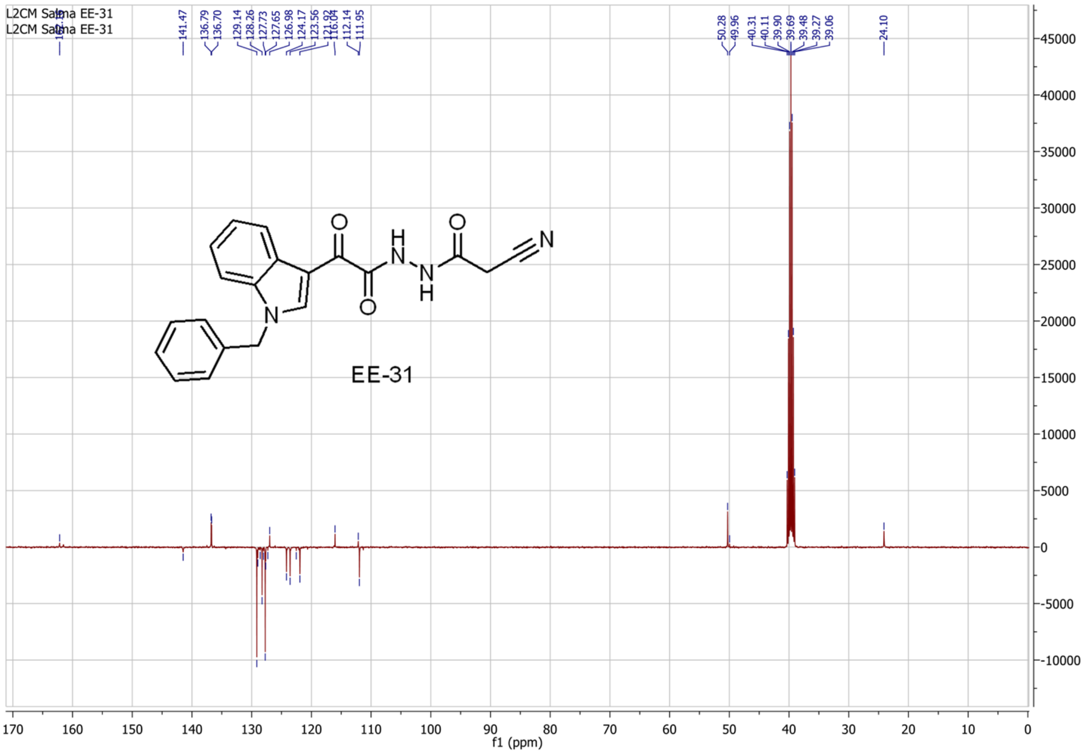

B. ^{13}C NMR spectrum of EE-80.

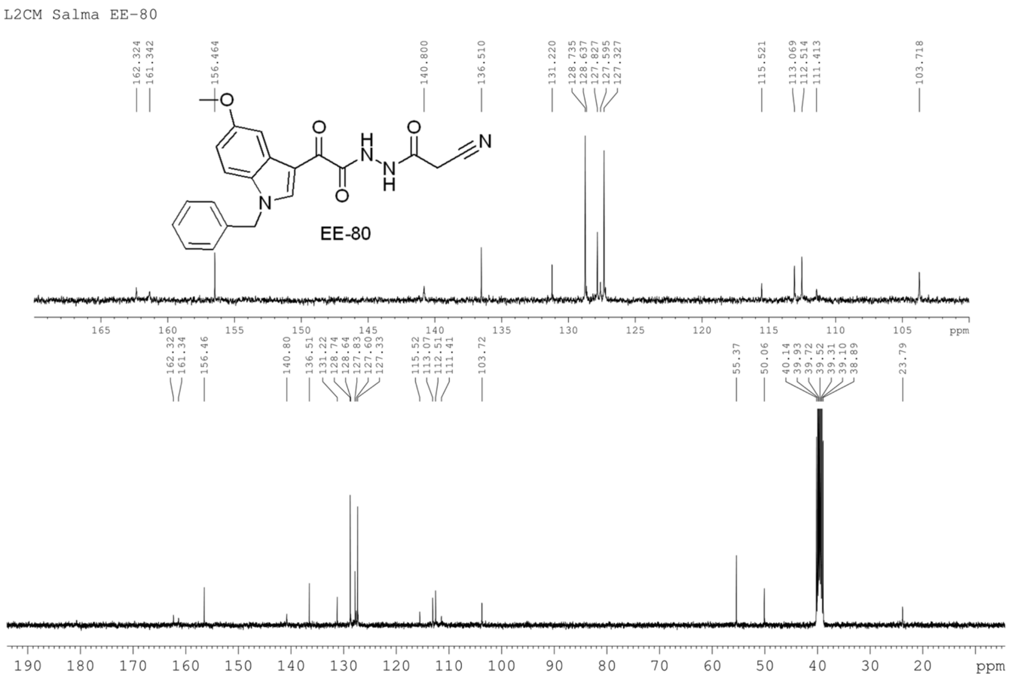

C. ¹³C NMR spectrum of EE-84.

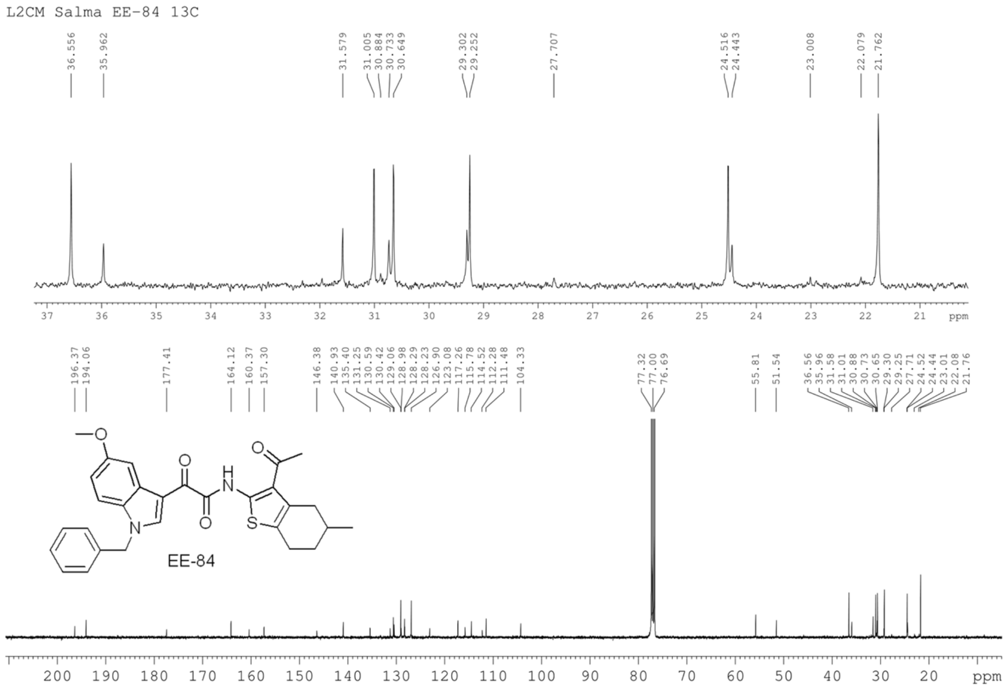

D. ¹³C NMR spectrum of EE-92.

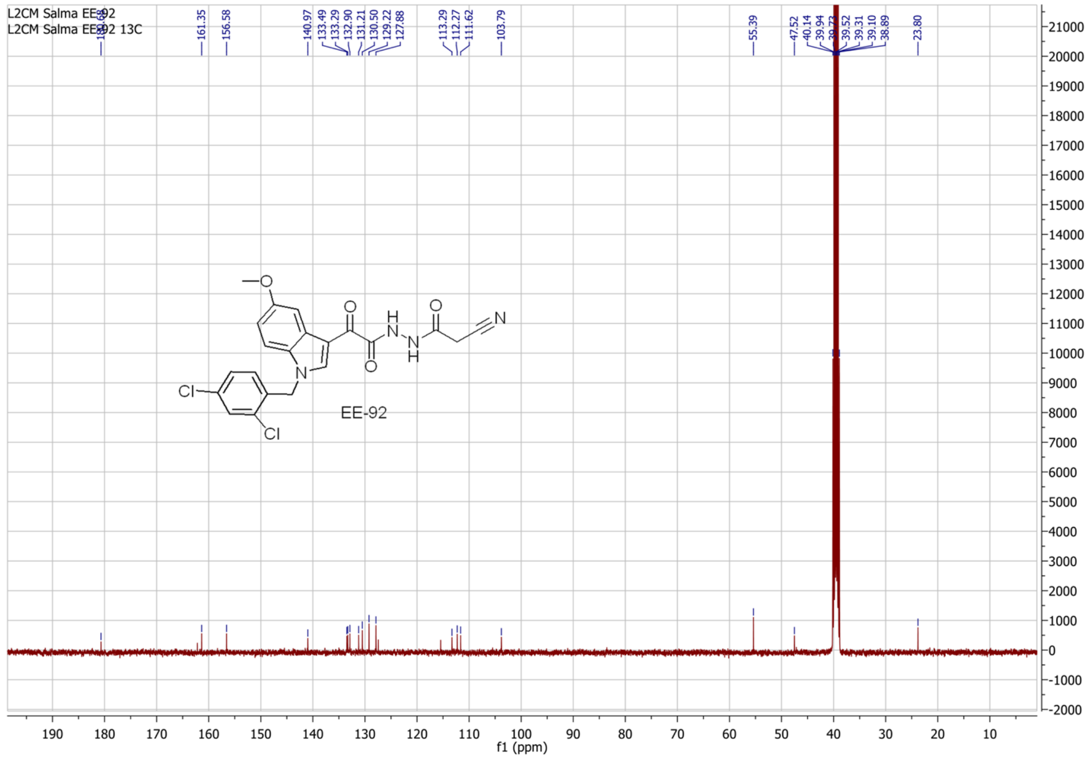

Supplementary Figure 3: ¹³C NMR spectral data of (A) EE-31, (B) EE-80, (C) EE-84, and (D) EE-92.

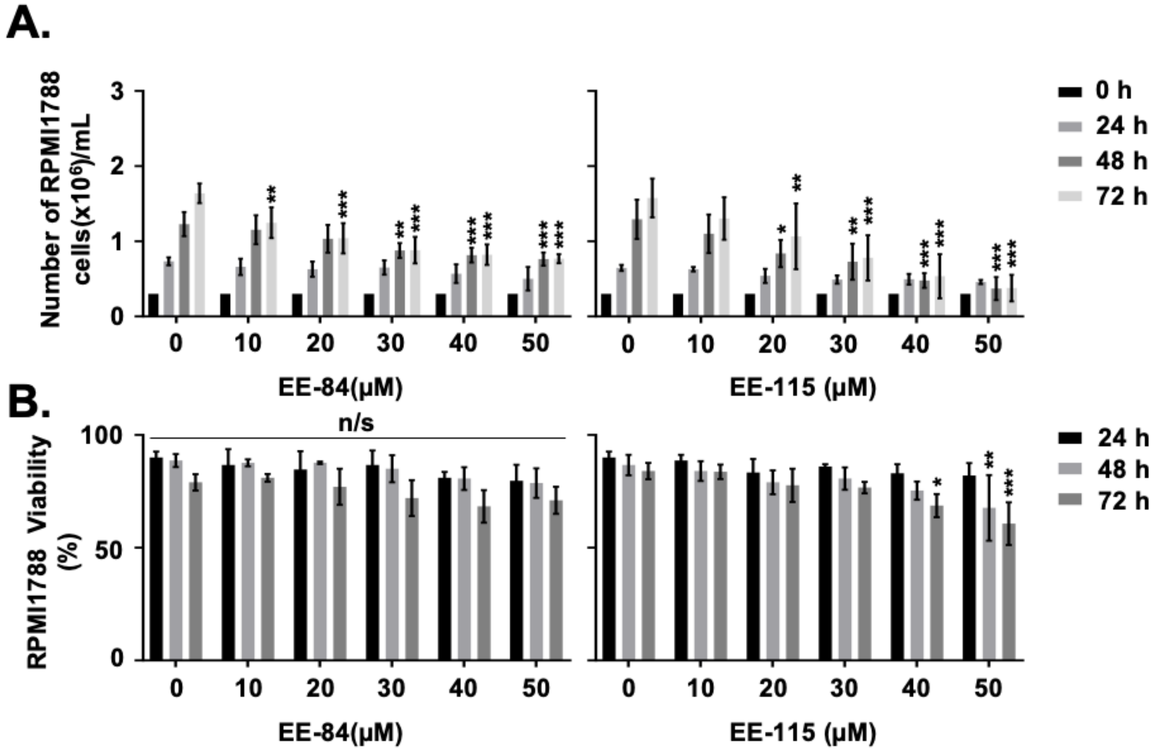

Supplementary Figure 4: Effect of EE-84 and EE-115 on proliferation and viability of non-cancerous cell line RPMI1788. (A) Proliferation and (B) viability of aplysinopsin-treated RPMI1788 were assessed by trypan blue exclusion test. Trypan blue exclusion test data represents the mean ± SD of three independent experiments. Statistical analysis: two-way ANOVA with Dunnett's multiple comparison test (trypan blue exclusion test); *p<0.05, **p<0.01, ***p<0.001 compared to controls.

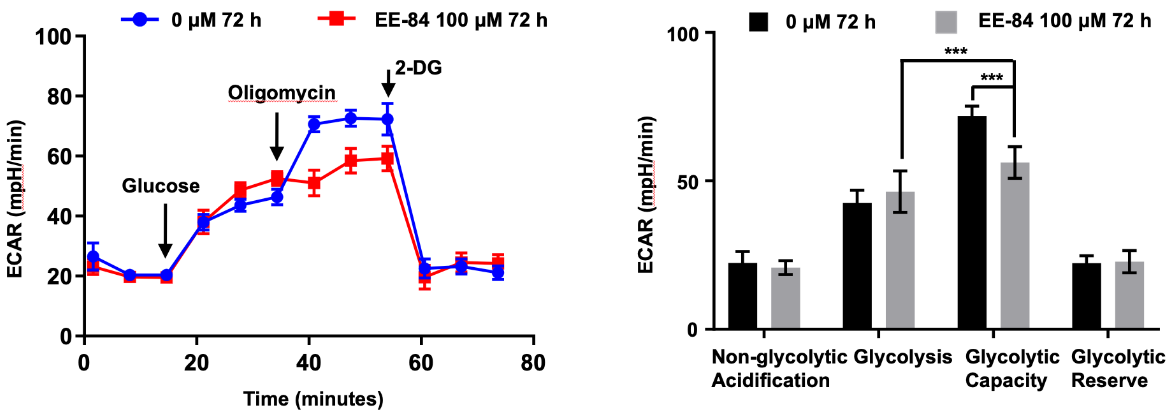

Supplementary Figure 5: EE-84 induced glycolytic stress on K562IR. (A) Measurement of glycolytic capacity in K562IR cells. 2-DG: 2-deoxy-D-glucose. The experiments were conducted using the Seahorse XFp Glycolysis Stress test, and the flow chart and bar graph show the measurement of ECAR. Results are the mean ± SD of three independent experiments. Statistical analysis was performed by two-way ANOVA, followed by Sidak's multiple comparisons test (Seahorse XFp Glycolysis Stress test) ***$p<0.001$ compared to controls.
